# A mutation in the melon Vacuolar Protein Sorting 41prevents systemic infection of *Cucumber mosaic virus*

**DOI:** 10.1038/s41598-017-10783-3

**Published:** 2017-09-05

**Authors:** Ana Giner, Laura Pascual, Michael Bourgeois, Gabor Gyetvai, Pablo Rios, Belén Picó, Christelle Troadec, Abdel Bendahmane, Jordi Garcia-Mas, Ana Montserrat Martín-Hernández

**Affiliations:** 1 Centre for Research in Agricultural Genomics (CRAG) CSIC-IRTA-UAB-UB, C/Vall Moronta, Edifici CRAG, Bellaterra (Cerdanyola del Vallés), 08193 Barcelona, Spain; 2 COMAV, Institute for the Conservation and Breeding of Agricultural Biodiversity, Universitat Politècnica de València (UPV), Camino de Vera s/n, 46022 Valencia, Spain; 3 INRA-CNRS, UMR1165, Unité de Recherche en Génomique Végétale, Evry, France; 4 IRTA (Institut de Recerca i Tecnologia Agroalimentàries), Barcelona, Spain; 5 Present Address: Unidad de Genética, Departamento de Biotecnología-Biología Vegetal, Escuela Técnica Superior de Ingenieros Agrónomos, Universidad Politécnica de Madrid, Madrid, Spain; 6 Present Address: KWS SAAT SE Grimsehlstr. 31, 37555 Einbeck, Germany; 7 Syngenta España S.A., C/Cartabona 10, 04710 El Ejido, Spain

## Abstract

In the melon exotic accession PI 161375, the gene* cmv1*, confers recessive resistance to *Cucumber mosaic virus* (CMV) strains of subgroup II. *cmv1* prevents the systemic infection by restricting the virus to the bundle sheath cells and impeding viral loading to the phloem. Here we report the fine mapping and cloning of *cmv1*. Screening of an F2 population reduced the *cmv1* region to a 132 Kb interval that includes a *Vacuolar Protein Sorting 41* gene. *CmVPS41* is conserved among plants, animals and yeast and is required for post-Golgi vesicle trafficking towards the vacuole. We have validated *CmVPS41* as the gene responsible for the resistance, both by generating CMV susceptible transgenic melon plants, expressing the susceptible allele in the resistant cultivar and by characterizing *CmVPS41* TILLING mutants with reduced susceptibility to CMV. Finally, a core collection of 52 melon accessions allowed us to identify a single amino acid substitution (L348R) as the only polymorphism associated with the resistant phenotype. *CmVPS41* is the first natural recessive resistance gene found to be involved in viral transport and its cellular function suggests that CMV might use CmVPS41 for its own transport towards the phloem.

## Introduction

Plant viruses are considered to be responsible for half of the emerging infectious diseases in plants^1^. Moreover, they can cause complete harvest lost and substantial economic losses, when they affect crops (reviewed by ref. 2). The use of naturally resistant cultivars is one of the most successful control measures against viral infections. While introgression, selection and breeding have been used for many years to develop resistant cultivars, nowadays the identification of the host factors governing resistance is providing potential targets for improving viral disease management.

About half of the approximately 200 known virus resistance genes in plants are recessively inherited^3^. Considering that recessive resistance seems to be more durable than dominantly inherited resistance^4^, focusing research on the genes involved provides a key tool to control plant diseases. The vast majority of the recessive resistance genes in plants identified to now encode eukaryotic translation initiation factors (eIFs), implicated in viral translation, but also in viral replication and in some cases, in cell-to-cell movement (for a review see refs 5 and 6). Significant advances have been recently achieved in the identification of host factors different than eIFs as naturally occurring recessive resistance genes in different pathosystems. For example, in barley, a protein disulfite isomerase-like (HvPDIL5-1), involved in protein folding, is encoded by the gene *rym11* and confers resistance to Bymoviruses^7^. In *Arabidopsis thaliana*, *rwm-1* encodes a phosphoglyceratekinase implicated in *Watermelon mosaic virus* accumulation^8^. As eIFs have been used in several plant species to identify or develop new resistant sources^6, 9–12^, identifying host factors other than eIFs involved in other steps of the viral cycle will provide new insights to understand plant-pathogen interactions and to identify new targets to develop resistant cultivars.

*Cucumber mosaic virus* (CMV), the type member of the Cucumovirus genus, has the broadest host range among viruses, being able to infect more than 1,200 species, including important crop plants within the Solanaceae, Cruciferae and Cucurbitaceae families, where they can cause severe damage worldwide^13^. Such extended host range involves an important genomic variability in CMV, which results in a high number of strains, usually classified into two subgroups, I and II, on the basis of their sequence^14^. Genetic control of resistance to CMV is heterogeneous, including dominant, recessive, monogenic and polygenic controls. The most common is recessive and polygenic and results in an impairment of long-distance movement of the virus^15–18^. In melon (*Cucumis melo* L.), studies evaluating a high number of accessions from different geographical origins reported three main strain-specific sources of resistance against CMV: “Freeman´s Cucumber” melon, the Japanese accession C-189, and the Korean accession PI 161375 cultivar “Songwhan Charmi”(SC)^19–22^. The CMV resistance present in SC was described as recessive, oligogenic^21^ and quantitative, with several QTLs involved and a major QTL mapping in linkage group XII (LGXII)^23^. Further studies demonstrated that *cmv1*, a single gene mapping in LG XII was able to confer by itself total recessive resistance to CMV subgroup II strains^24, 25^. Besides, at least two additional QTLs (*cmvqw3.1* and *cmvqw10.1*) must act together and cooperatively with *cmv1* to confer resistance to the more aggressive subgroup I strains^26^.

Reassortants and chimaeras between CMV-FNY (subgroup I) and CMV-LS (subgroup II) revealed that the determinant of the virulence against *cmv1* was the movement protein (MP). Moreover, a combination of four residues or group of residues in the MP of CMV-FNY was identified as the only requirement of the MP to overcome *cmv1*-mediated resistance^25^. CMV-LS, was able to replicate and move cell-to-cell in the resistant plant. However, it was never detected in the phloem. Immunogold labeling experiments established that the virus was present in the bundle sheath (BS) cells of the resistant plant, but never reached the vascular parenchyma (VP) cells or the Intermediary cells (IC)^27^. Therefore, *Cmv1/cmv1*allows or prevents systemic infection by acting as a molecular gate, only in the bundle sheath (BS) cells, and determining the entrance of the virus in the phloem in a manner dependent on the viral MP present. Thus, *cmv1* represents a new host factor implicated on a poorly studied step of viral infections, the transport from mesophyll to phloem to establish a systemic infection.

In the present study, we have first narrowed the interval carrying *cmv1* from 2.2cM^24^ to a region of 132 Kb and demonstrate that *cmv1* encodes a Vacuolar Protein Sorting 41 (VPS41), a protein involved in vesicle trafficking from late Golgi to the vacuole. We show that the transgenic expression of the susceptible Piel de Sapo (PS) allele of *CmVPS41* in the SC resistant background is able to restore the infection by CMV and that a TILLING mutant in the *CmVPS41*gene impairs CMV-LS infection. Besides, a collection of 52 melon accessions was used to identify the causal polymorphism in the *CmVPS41* gene. This is the first cloning of a natural recessive resistance gene involved in a new mechanism of resistance based on blocking long distance viral transport by preventing virus entry into the phloem.

## Results

### Fine mapping of *cmv1*

**Figure 1:**
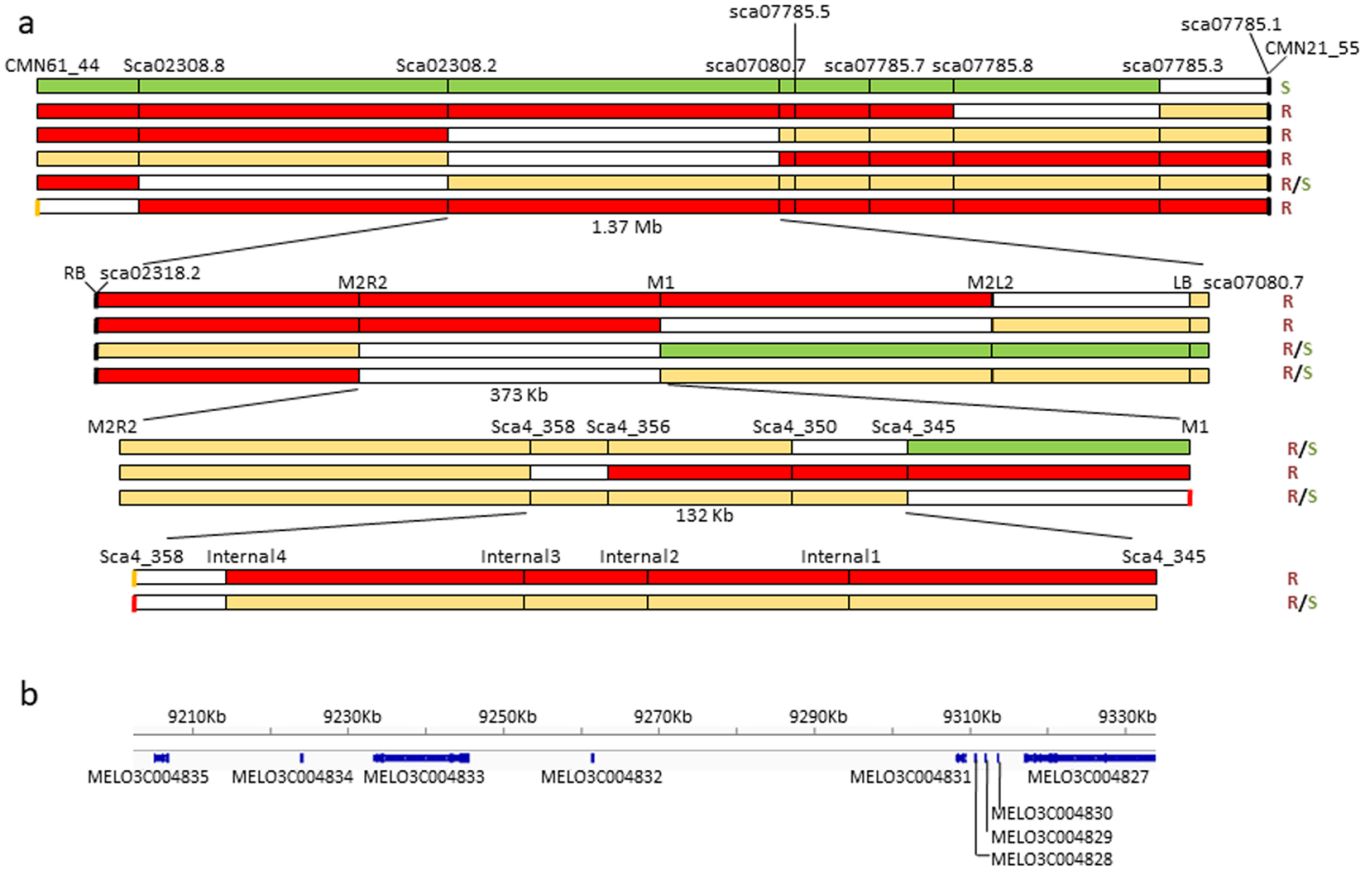
Fine mapping of *cmv1*. (**a**) Sequential intervals screened during fine mapping. Each horizontal bar represents a group of recombinants inside the region, colors indicate the origin of each region. Red, SC; green, PS; yellow, heterozygous and white, recombination interval. The phenotype of the recombinants is noted at the right. R, S and R/S for resistant, susceptible and segregating F3 plants. (**b**) Annotated features inside the smallest *cmv1* interval.

The melon *cmv1* gene, which confers total resistance to CMV strains from subgroup II^25^, was located in LG XII within a 5.78 Mb interval flanked by markers CMN61_44 and CMN21_55^24^. Fine mapping of *cmv1* was addressed using an F_2_ population from a cross between the susceptible accession PS and the NIL SC12-1, which carries the resistant allele^24^. A first set of 780 F_2_ individuals, screened with the above flanking markers, allowed identifying 55 recombinant individuals. Genotyping of those individuals with eight SSR markers distributed in the 5.78 Mb interval (Fig. 1a, Supplementary Table S1) identified five different recombination regions. Inoculation of F3 plants with CMV-LS, mapped *cmv1* within an interval of 1.37 Mb flanked by SSR markers Sca02308.2 and Sca07080.7. The 14 recombinants lying within this 1.37 Mb interval were then genotyped with internal markers, defining a 373 Kb interval, from marker M2R2 to M1. Finally, three out of 14 recombinants allowed reducing the *cmv1* interval to 132Kb between markers Sca4_345 and Sca4_358. Since no further recombinants in this interval were available, we carried out a screening of a new set of 3455 F_2_ individuals that finally rendered only three additional recombinants between those markers. The genotyping of these individuals with internal markers showed that their recombination breakpoints were located between markers Sca4_358 and Internal 4. Subsequent phenotyping of the corresponding F3 progeny did not allow us to further reduce the *cmv1* interval (Fig. 1a).

### Functional analysis of *cmv1* interval and candidate gene identification

**Figure 2:**
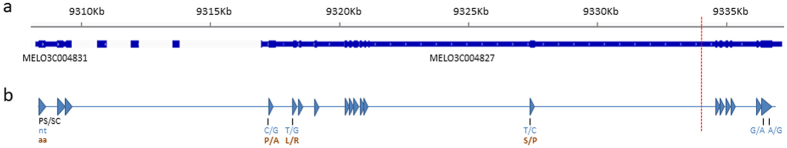
Structure of *CmVPS41* gene. (**a**) *CmVPS41* structure according to annotation on the melon genome. Both genes MELO3C004831 and MELO3C004827 correspond to a single open reading frame. The genomic DNA expands 28.9Kb. (**b**) *CmVPS41* gene structure based on the complete coding sequence. The 19 exons produce a 2,883 bp cDNA. SNPs between PS and SC are detailed below in blue. In red, the amino-acid changes in exons 4, 5 and 13. The red line demarks one of the ends of the *cmv1*interval.

According to the available *in silico* annotations of the melon genome, the 132 Kb *cmv1*interval contains eight predicted genes and part of the MELO3C004827 gene (Fig. 1b, Supplementary Table S2). Five of them correspond to sequences smaller than 500 bp, not validated by ESTs. Thus, they are unlikely functional genes. The four remaining genes are MELO3C004835, encoding a possible Lysosomal Pro-X carboxypeptidase (PCP), MELO3C004833, encoding a Crooked neck-like protein 1 (CNL) and two annotated genes, MELO3C004831 and MELO3C004827, that correlate with the C-terminal and N-terminal part, respectively, of a Vacuolar protein sorting-associated protein 41 (VPS41). Analysis of the polymorphisms between both parental lines PS and SC in the 132 Kb *cmv1* interval^28^ revealed no variable transposon insertions or structural variations. However, they detected 1,318 polymorphisms including SNPs and INDELs. From those polymorphisms, 283 lay inside or close (1 kb up/downstream) to the *PCP*, *CNL* or *VPS41* genes, and could modify their function. We detected 10 SNPs inside the exons of the three genes, although only five of them were non-synonymous: one in *PCP*, one in *CNL* and three in MELO3C004827 *VPS41*. When the predicted *VPS41* ORFs were manually curated, the results strongly suggested that both predicted genes corresponded to a single ORF whose genomic sequence would span 28.9Kb and would have 19 exons (Fig. 2 and Supplementary Table S3). Cloning of the complete *VPS41* cDNA from both PS and SC lines confirmed that, indeed, it was a unique ORF of 2,883 bp. Thus the *cmv1* interval includes 3 likely genes and five non-synonymous polymorphisms between PS and SC that might be responsible for the resistance to CMV. Considering that *cmv1* blocks the movement of the virus from the BS cells to the phloem cells^27^, our main candidate gene was *CmVPS41*, since it is involved in the transport of vesicles and cargo proteins towards the vacuole^29, 30^ and this function could be recruited by the virus for its own transport. In fact, VPS41 has been implicated in animal virus release from the infected cell^31^.

### *cmv1* encodes a Vacuolar Protein Sorting 41

**Figure 3:**
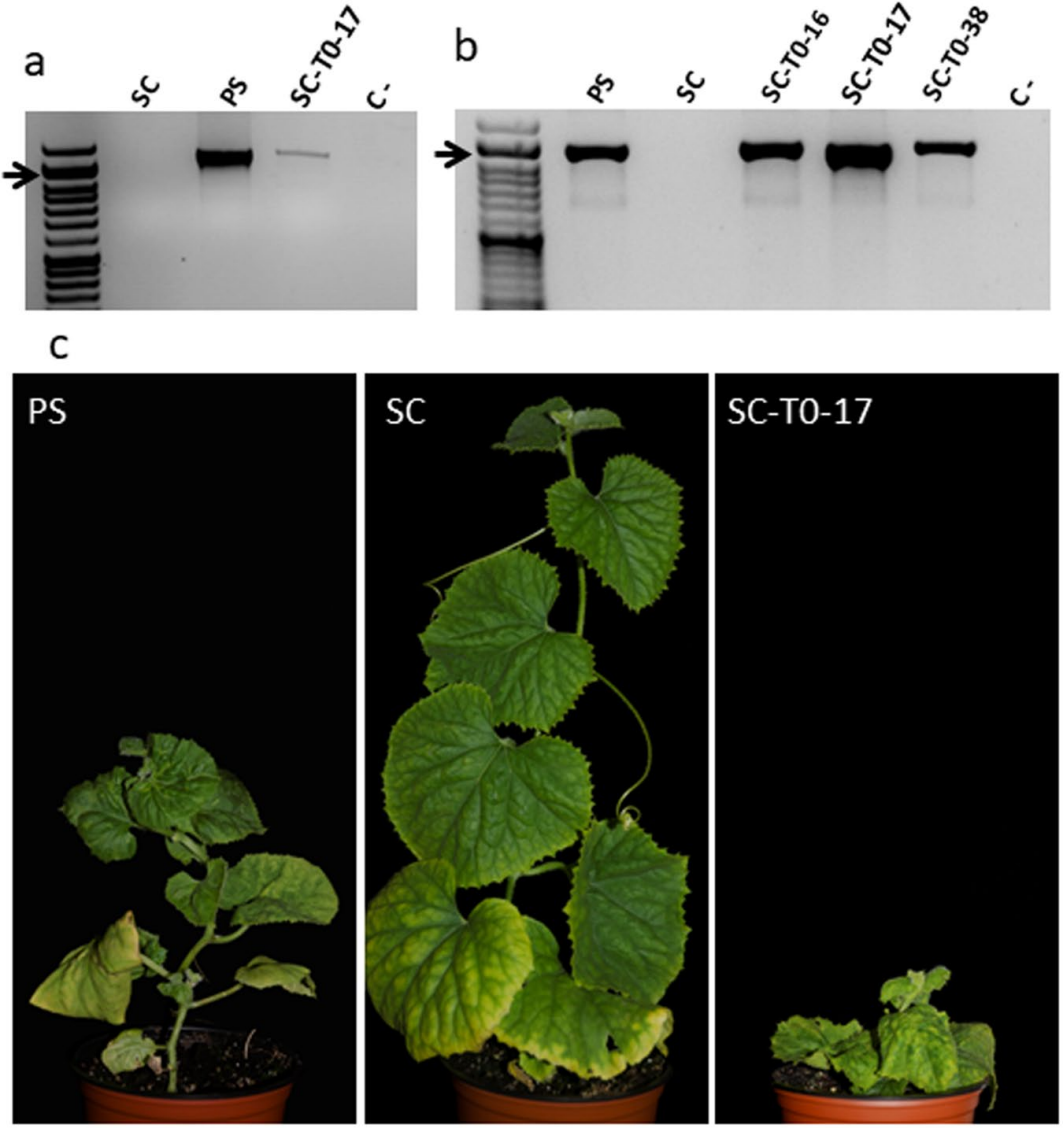
CMV infection of T0 transgenic plants expressing PS *CmVPS41*. Representative results 21 days after the inoculation with either CMV-LS or CMV-FNY are shown (**a**). RT-PCR detection of CMV-LS-infected plants. (**b**) RT-PCR results after inoculation with CMV-FNY. (**c**) *In vitro* acclimatized T0 transgenic plants inoculated with CMV-FNY. PS: non-transgenic, *in vitro* regenerated susceptible line; SC: non-transgenic, *in vitro* regenerated resistant line; SC-T0: transgenic SC lines (16, 17 and 38) expressing *CmVPS41* from PS under its own promoter. C- negative RT-PCR control. Arrows indicate the 1200 bp band of the size marker.

Validation of *CmVPS41* as the *cmv1* gene was carried out by two complementary approaches. First, we generated transgenic susceptible melon plants expressing the dominant PS susceptible allele in the resistant SC background. As a second approach, we searched for *CmVPS41* mutants with enhanced resistance to CMV-LS in a susceptible background. The susceptible PS allele was cloned under its own promoter and transformed into the SC resistant accession to generate three independent lines expressing the gene (Supplementary Figure S1). Given that *cmv1*is sufficient to confer resistance to CMV-LS, but also necessary for resistance to CMV-FNY^25, 26^, expression of the PS allele of *CmVPS41* should allow both CMV-LS and CMV-FNY to spread in the transgenic plants. The three transgenic T0 lines were tetraploid, a frequent event in melon transformation^32, 33^. Thus, as we could not obtain diploid lines after self-pollination, we directly used the T0 generation for phenotyping. *In vitro* replicated T0 clones from the three independent transgenic lines were newly rooted, acclimated and then inoculated with either CMV-LS or CMV-FNY. CMV-LS infection was detected as a mosaic only in one line (SC-T0-17), two leaves above the inoculated leaves and was subsequently confirmed by RT-PCR using specific primers from CMV-LS (Fig. 3a). However, inoculation with CMV-FNY produced mosaic symptoms in all plants from the three transgenic lines, whereas the untransformed *in vitro* regenerated SC control plants remained symptomless and untransformed *in vitro* regenerated PS control plants were systemically infected (Fig. 3c). Infection was confirmed by detection of CMV-FNY by RT-PCR using specific primers from CMV-FNY (Fig. 3b). Thus, the results show that the expression of the susceptible *CmVPS41*allele in the resistant SC accession allows CMV systemic infection and, therefore, that CmVPS41 protein is indeed encoded by *Cmv1*. The limited infection by CMV-LS suggests that as the transgenic lines are tetraploid, and they may carry only one copy of the susceptible allele, the limited amount of PS CmVPS41 protein could be a limiting factor for CMV-LS infection.

**Figure 4:**
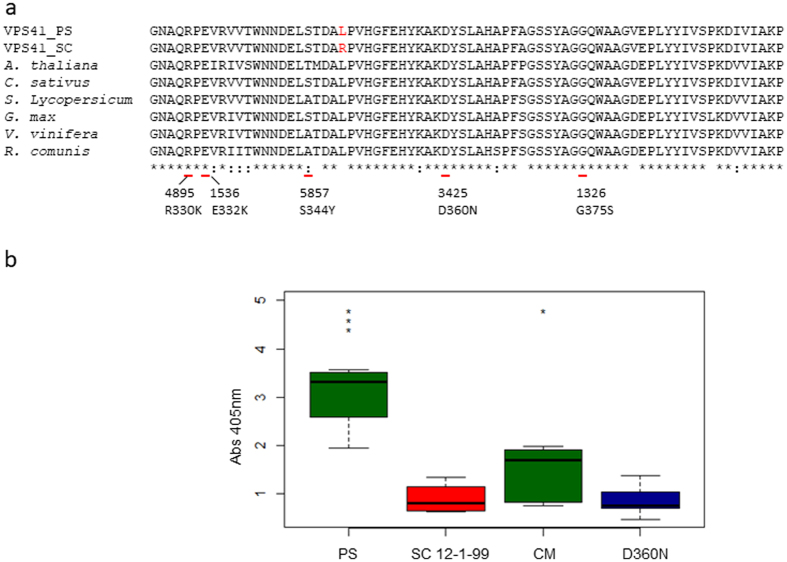
(**a**) Amino acid sequence of the *CmVPS41* fragment screened in the TILLING population (exons 5 and 6). The sequence was aligned against the corresponding sequence from representative plant species. Detailed below are the nonsynonymous TILLING mutants detected. (**b**) CMV-LS accumulation in the inoculated D360N TILLING family. Samples were collected after 21dpi. ELISA results from the fourth true leaf from a representative experiment are shown. PS, Piel de Sapo susceptible line; SC12-1-99, introgression line carrying *cmv1*; CM, CharMono susceptible TILLING parental line; D360N, TILLING family carrying the mutation D360N in *CmVPS41*. *p-value for the t-test comparing against M3425 lower than 0.05, **lower than 0.01, ***lower than 0,001.

Screening of 6,200 M2 families of a TILLING population developed on the CharMono background^34^, a melon accession susceptible to CMV, identified 14 mutants, with six of the mutations lying within the coding sequence (Fig. 4a and Supplementary Table S4). One of the mutations was synonymous, two of them produced amino acid changes predicted as neutral by PROVEAN software and three were predicted as deleterious. The TILLING mutants were subjected to three rounds of self-pollination selecting for the five non-synonymous mutations in *CmVPS41* to get homozygous plants and to prevent the presence of contaminating mutations in other parts of the genome related to the phenotype. After challenging plants of the five families carrying non-synonymous substitutions with CMV-LS, all of them showed infected plants, indicating that none of the mutations conferred total resistance to the virus. However, mutant plants carrying the mutation D360N showed milder symptoms than CharMono, the susceptible control parental line of the TILLING population. Moreover, after 21 dpi, the plant upper leaves of the mutant D360N seemed to recover, unlike PS and CharMono susceptible control plants. CMV detection in the fourth leaf of each plant at 21 dpi showed that, while in some of the mutant plants the virus was still present, the viral load was significantly lower when compared with PS (p-value = 9.306e-06) and CharMono (p-value = 0.0379) plants (Fig. 4b). The SC 12-1-99 subNIL resistant control carrying *cmv1* remained uninfected. The experiment with the mutant D360N was repeated three times, inoculating 10 plants in each experiment, with similar results. Interestingly, the mutation D360N was predicted as the most deleterious for the CmVPS41 protein of all TILLING mutations found (Supplementary Table S4). Thus, these results indicate that mutations in the *CmVPS41* gene lead to lower susceptibility to CMV-LS, confirming that *CmVPS41* is encoded by *cmv1*.

### Identification of a mutation associated with the resistant phenotype by the analysis of a diverse collection of melon accessions

Out of the three non-synonymous changes between PS and SC found in exons 4, 5 and 13 of *CmVPS41* (Fig. 2), only the substitution L348R at exon 5 is predicted as deleterious for the protein by PROVEAN and MutPred software (Supplementary Table S5), whereas the substitutions P262A and S620P should be neutral. This suggests that the change L348R could be the causal polymorphism of the resistant phenotype given by the SC allele of *CmVPS41*. To confirm this hypothesis, we genotyped *CmVPS41* in a collection of 52 diverse melon accessions^35, 36^ with molecular markers developed to detect the three SNPs (Supplementary Table S1) and studied the correlation of each mutation with the susceptibility or resistance to CMV-LS. The collection included 41 accessions of *C. melo ssp.melo* and 11 *C. melo ssp. agrestis* (Supplementary Table S6). Most of the accessions carried the SC allele at exons 4 and 13, and the PS allele at exon 5.The PS haplotype was shared by only four accessions from the *cantalupensis* group, all French varieties. Can-PMRUSA a *reticulatus* type, and Am-Korsa-Rus an *ameri* type, carried PS alleles at exons 5 and 13, and SC allele at exon 4. Con-GMJa, an Asiatic melon belonging to the *conomon* type, was the only accession that shared SC haplotype and carried the SC allele at exon 5 (Supplementary Table S6). A subset of nine melon accessions carrying all observed haplotypes were selected for phenotyping: five with the most frequent haplotype (In-Kirk-Turk, In-Ps-PipaSp, Mom-PI124Ind, Ag-Tri Ind and Mom-MR1Ind) two with PS haplotype (Can-Ved Fran and Can-NC Fran), the one with SC haplotype (Con-GM Ja) and one with SC allele only at exon 4 (Can-PMR USA) (Table 1). After challenge with CMV-LS, all of them were systemically infected except Con-GM Ja, the only one carrying the SC allele at the SNP in exon 5 (Table 1). Thus, the only SNP associated with the resistance was located in *CmVPS41* exon 5, suggesting that the mutation L348R is the causal polymorphism for the resistance given by *cmv1*. Accordingly, real-time experiments to monitor gene expression between PS and SC showed no significant variations in leaf, vein and petiole tissues of both parental lines (Supplementary Figure S2), confirming that the causal mutation was located in the coding sequence and not in regulatory regions.

**Table 1:** Response of diverse *C. melo* accessions to CMV-LS inoculation.

Code	Comon name	*CmVPS41*			CMV-LS
		G/C Exon 4	G/T Exon 5	C/T Exon 13	
Can-VedFra	Vedrantais	PS	PS	PS	S
In-Kirk Turk	Kirkagac	SC	PS	SC	S
Can-NCFra	Noir des carmes	PS	PS	PS	S
In-PsPipaSp	Pipa de Oro	SC	PS	SC	S
Can-PMRUSA	PMR45	SC	PS	PS	S
Mom-PI124Ind	PI 124112 (Calcuta)	SC	PS	SC	S
Ag-TriInd	Trigonus	SC	PS	SC	S
Mom-MR1Ind	MR1	SC	PS	SC	S
Con-GMJa	Ginsen makuwa	SC	SC	SC	R

PS, Piel de Sapo allele; SC, Songwhan Charmi allele; R, resistant; S: susceptible.

### *VPS41* gene is present as a single gene in most plant species

*VPS41* gene is part of the “Homotypic Fusion and Vacuole Protein Sorting” (HOPS) complex, which is present in a wide range of species from animals to yeast^37^. A search in the PLAZA 3.0 database (http://bioinformatics.psb.ugent.be/plaza/), which contains data of plant genomes that have been fully sequenced, shows that *CmVPS41* belongs to the orthologue family *ORTHO03D004047*. This gene is present in 29 species out of 31 included in PLAZA 3.0 and in 22 of these species the gene is present as a single copy (Supplementary Table S7), as it is the case in *C. melo*. In these species, VPS41 might also have a role in CMV transport in the susceptible species. The coded protein, contains a Clathrine domain, characteristic of many proteins involved in vacuolar maintenance and protein sorting^38^, and a WD-40 domain, presumably involved in protein-protein interaction. The causal polymorphism L348R is outside both of them and is located in a highly conserved region of the gene (Fig. 4a, Supplementary Figure S3). All species, including *C. melo*, have a Leucine at position 348 except the resistant melon accession SC, which has an Arginine. The location and identity of the mutation L348R in this conserved region, where only one TILLING mutant (D360N) showed a mild effect on CMV resistance, suggests that this could be the key amino-acid for the interaction with CMV and suggests the possibility of testing the effect of this mutation in other species susceptible to CMV.

## Discussion

In melon, *cmv1* confers recessive resistance to CMV strains of subgroup II. It is also necessary to confer resistance to subgroup I strains. However, in this case, the cooperation of other two QTLs with *cmv1*is also required^25^. Here we have identified CmVPS41, a protein involved in membrane trafficking to the vacuole as part of the “Homotypic Fusion and Vacuole Protein Sorting” (HOPS) complex, as the product of *cmv1*. We have validated *CmVPS41* as *cmv1* both by generating transgenic susceptible lines expressing the susceptible allele in the resistant parental line, and by characterizing a TILLING mutant with reduced susceptibility to CMV-LS.

The HOPS complex is involved in regulating the vesicle transport from the late endosome, but also directly from the Golgi complex, through the AP-3 pathway, to the vacuole^37^. It is a multiprotein complex composed of six subunits. Four of them constitute the core proteins and are shared with CORVET, another complex guiding endosome life cycle. The remaining two subunits, VPS41 and VPS39, are specific for HOPS and confer the tethering function that enables HOPS to promote vacuolar fusion, being VPS41 the effector subunit^38–41^.

Our previous studies have determined that *Cmv1/cmv1* is a checkpoint for CMV phloem entry, acting only in the BS cells. The susceptible allele *Cmv1*would permit virus transport from BS cells to VP cells or ICs to develop a systemic infection, whereas the resistant allele *cmv1* would prevent this movement^27^. According to our data, a single amino acid change (L348R) in the CmVPS41 protein would modify the putative interaction with the CMV movement protein (MP), the viral interacting factor that determines the onset of the systemic infection^25^. Therefore, in the resistant plant, this putative interaction would not happen. However, a set of four simultaneous mutations in the MP of the virus are able to restore the systemic infection in the resistant plant^25^, suggesting that these changes are able to restore the putative interaction with CmVPS41encoded by the resistant plant as well. Altogether, this suggests that, in the susceptible plant, CMV could be recruiting some CmVPS41 activity either to mediate its own transport towards the PDs or alternatively, it could be needed already in the PDs to gate them for CMV movement to the phloem. In this scenario, the main activity of CmVPS41 in vesicle fusion would remain unchanged, since both resistant and uninfected susceptible plants have the same phenotype. Likewise, *zip-2*, an Arabidopsis missense mutant in *AtVPS41* in a highly conserved amino acid has no physiological or morphological phenotype but is able to suppress *zig-1*, a mutant impaired in membrane trafficking towards the vacuole. It has been suggested that the *zip-2* mutation alters part of AtVPS41 function, but the essential function remains intact^42^. The inability to obtain null mutants from a T-DNA insertion collection strongly suggests lethality in homozygosis of AtVPS41 mutants. In a similar way, in our TILLING screening in the *CmVPS41*gene, we did not identify any stop codon mutant, supporting again an essential function of CmVPS41 for the cell. In fact, out of the five mutants identified presenting amino acid change, only one showed an enhanced (but not fully) resistant phenotype. The rest did not show any altered phenotype. This is expected for a gene with an essential function that cannot be easily modified without strongly compromising plant viability.

Recessive resistance genes, like *cmv1*, are host factors recruited by the virus to complete their cycle, and thus they can be exploited to protect plants from viral infections. Most of the natural recessive resistance genes identified and cloned to date encode translation initiation factors (eIFs)^5, 43^ and are involved in virus replication. Since first identified in pepper^44^, their key role in viral infections has also been demonstrated in more than 20 plant species and 30 viruses and have been exploited as targets to produce plants resistant to a broader spectrum of viruses in several crop species (reviewed by Sanfaçon, 2015). Only a few examples of natural recessive resistance involve genes other than eIFs, although they also restrict virus multiplication^7, 8^. *cmv1* is the first natural recessive resistance gene whose function has been located in a particular cell type, where it is involved in the transport of the virus to the phloem to produce a systemic infection^27^. Since *cmv1* is able to block CMV-LS in the BS cells and VPS41 is involved in intracellular membrane trafficking, this suggests that CmVPS41 protein is involved in CMV transport in the boundary BS cells/phloem, rather than in viral replication. Our findings suggest a new mechanism for a natural recessive resistance whose molecular characterization could provide a whole new range of plant virus control strategies targeting either CmVPS41 or other participants in the same step of CMV systemic transport. Furthermore, the identification of the causal mutation in *CmVPS41* enables the directed gene editing by CRISPR/Cas9 to generate melon resistant plants and this possibility could also be explored in the VPS41 gene from other crops.

To date, while no other VPS has been demonstrated to be involved in a natural virus resistance, VPSs and other components of the vesicle trafficking machinery have been implicated in virus life cycle. Most of both animal and plant viruses associate to the host secretory pathways components for different purposes: to accomplish replication, but also for movement and budding^45, 46^. In animals, enveloped viruses exploit the endomembrane system to enter their hosts^47^. The VPS pathway is necessary for the release of Marburg virus through VPS4^31^, whereas hVPS41 is increasingly associated with human pathologies and together with other HOPS components, was shown to be essential for Ebolavirus infections^48^. Several plant viruses are known to interact with components of membrane trafficking complexes on its own benefit, therefore facilitating the transport and replication of the viral material. For example, the Tombusvirus *Tomato bushy stunt virus* (TBSV) interacts through its p33 protein with VPS23 and Vps4p, two members of ESCRT, another complex involved in endosome life cycle mediating vesicle formation^49^. VPS23 is also the interacting protein for *Carnation Italian ringspot virus* (CIRV) p36, involving the ESCRT complex with viral replication^50^. COP II, the complex that regulates vesicle trafficking between ER and GA, is also involved in replication of *Turnip mosaic virus* (TuMV) through its 6K2 protein^51^ and of MNSV replication, through its p/B protein^52^. All the above examples involve interactions between viral and host proteins found by different methods, but not natural resistances against the viruses involved, suggesting again that mutations in genes encoding components of vesicle trafficking machinery can often be deleterious for the plant. Only in cucumber, a VPS4 was recently proposed, although not validated, as a candidate for the *zym1*gene that confers resistance to *Zucchini Yellow Mosaic Virus* (ZYMV)^53^. As VPS4 is often recruited as a permanent component of the viral replication complexes, (VRCs), it could be likely affecting ZYMV replication.

Due to the nature of CmVPS41, our results support the idea that vesicular endocytosis and plasmodesmata may share some regulatory mechanisms regarding virus movement^54^. Further effort should be done to determine the molecular mechanism of the resistance conferred by this gene and its relation with the viral MP. The identification and cloning of *CmVPS41*allow the possibility to address new questions related to the precise mechanism of resistance such as how and where does the interaction between CmVPS41 and viral MP take place or how this interaction allows the virus to go through the PDs that communicate BS and phloem cells to produce a systemic infection. Moreover, since the participation of the CmVPS41 seems to be necessary only to go through these PDs, they could be different than those communicating BS cells with other mesophyll cells. Additionally, it remains to be seen if VPS41 is a general checkpoint for CMV phloem invasion in other species. Identification of *cmv1*also opens up possibilities as a first step to start pyramiding in melon elite cultivars all the QTLs involved in resistance to CMV once they are identified.

## Materials and Methods

### Plant and virus material

For fine mapping of the *cmv1* gene, a melon F2 population from the cross between the susceptible line T111 of the Spanish cultivar Piel de Sapo (PS) type and the resistant Near Isogenic Line (NIL) SC12-1 was used. The NIL SC12-1 carries a single introgression from the resistant Korean accession PI 161375, cultivar ‘Songwhan Charmi’ (SC) in LG XII containing the *cmv1* gene in the genetic background of PS^24^. F_2_ recombinant plants were selected using flanking molecular markers (see below) and self-pollinated to obtain the F3 plants used for phenotyping. The *C. melo* accessions representing a wide range of melon diversity^35, 36^ were provided by the Cucurbits group of the COMAV-UPV (Instituto de Conservación y Mejora de la Agrodiversidad Valenciana, Universidad Politécnica de Valencia, Spain). For the CMV resistance assays, PS was used as susceptible control and SC and the sub NIL SC12-1-99, which carries a shorter introgression in LG XII containing *cmv1* ^24^, were used as resistant controls. Negative controls were prepared from leaf tissue extracts of mock-inoculated plants. Virus strains used in this study were CMV-LS, belonging to subgroup II, and CMV-FNY, belonging to subgroup I, kindly provided by Dr Peter Palukaitis as infectious clones^55, 56^.

### Virus inoculation

For virus inoculation experiments, seeds were pre-germinated and grown as previously described^25^. Inoculation and virus detection were carried out as previously described by Essafi and collaborators^24^. Briefly, viral inocula were prepared from freshly symptomatic leaves of zucchini squash ‘Chapin F1’ (Semillas Fito SA, Barcelona, Spain) and rub-inoculated onto the cotyledons of young 7-10-day old melon plants. Symptoms were scored visually at 14, 21 and 28 days post-inoculation (dpi). For inoculation of *in vitro* acclimated transgenic plants, six newly rooted *in vitro* clones from each of the T0-SC independent lines, four PS and four SC control *in vitro* regenerated plants were transferred to soil pots inside individual plastic bags. Plants were kept in growth chambers (Sanyo MLR-350H, Sanyo Electric Biomedical Co, Osaka, Japan) under long-day conditions of 22 °C for 16 h with 5000 lx of light and 18 °C for 8 h in the dark during 3 days, inside plastic bags to keep high humidity, and then one day with the plastic bag open. Then, the plastic bag was removed and the plants were kept for four days longer in the chamber until two newly formed leaves were inoculated with CMV, as already described^25^.

### Virus detection

Virus detection by DAS-ELISA was carried out at 21 dpi from young developed leaves using CMV coat protein-specific polyclonal antiserum (Lowe Biochemica GmbH, Otterfing, Germany) following manufacturer´s protocol. ELISA reactions were measured spectrophotometrically after 60 minutes reaction at 405 nm in a VICTOR3 V multilabel plate reader (Perkin Elmer Inc., Waltham, MA, USA). Viral detection by RT-PCR was performed as described^25^. For CMV-FNY detection, specific primers F109-3′R (TGGTCTCCTTTTAGAGACCC) and F109-2200F (CGGGACCATTAGTCAAGTTG) were used to amplify a 1,147 bp fragment. For CMV-LS, specific primers LS1-1400R (GAAGCATTCCACATATCGTAC) and LS1-1F (GTTTTATTTACAAGAGCGTACG) were used to amplify a 1,400 bp fragment.

### Development of molecular markers

Molecular markers used for fine mapping of *cmv1* (SSRs and SNPs) and for *cmv1* analysis in diverse melon accessions are described in Supplementary Table S1. The flanking markers of the *cmv1* interval, SSRs CMN61_44 and CMN21_55^24^ had previously been developed^57^. The remaining markers were developed from polymorphisms detected by re-sequencing PS and SC lines^58^. The polymorphisms were selected based on their physical position in the melon genome and the sequence flanking the polymorphisms was used to develop the molecular markers (sequence available from http://melonomics.net). For SSRs markers, primers flanking the repetitive sequence were designed using Primer3^59^ (http://bioinfo.ut.ee/primer3-0.4.0/). One of the primers was labelled with IRD-800 (MWG Biotech AG, Ebersteg, Germany). After PCR amplification, fragments were visualized with LICOR IR2 sequencer (Li-corInc, Lincon, New England, USA) as described previously^60^. For SNPs genotyping, three methods were used: Sanger sequencing, Taqman probes and CAPS assay (SNPs/sanger, SNPs/Taqman and SNPs/CAPS). For SNPs/Sanger and Indel/Sanger sequencing markers, primers flanking the polymorphism were designed also with Primer3. PCR amplified fragments were sequenced by capillary electrophoresis at Macrogen (Macrogen Europe, Amsterdam, The Netherlands). Sequences were analyzed with Sequencher® version 5.0 sequence analysis software (Gene Codes Corporation, Ann Arbor, MI USA http://www.genecodes.com) and aligned with PS and SC sequences in order to determine the SNPs allele. Probes for SNPs/Taqman markers were ordered as Custom TaqMan® SNP Genotyping Assays (Applied Biosystems, Foster City, CA, USA) by submitting the SNP and flanking sequence. Genotyping was performed using a LightCycler 480 instrument (Roche Applied Science, IN, USA), mixing Taqman probes with LightCycler 480 Probe Master (Roche Applied Science, IN, USA) following manufacturer’s instructions. Genotype calling was done with the endpoint genotyping analysis procedure available from the LightCycler 480 software version 1.5. (Roche Applied Science, IN, USA). For SNPs/CAPS markers, primers flanking the polymorphism were designed also with Primer3. PCR amplified fragments were digested with the restriction enzyme, either *BsiEI* or *TseI* (New England BioLabs Inc., MA, USA) following manufacturer’s protocol. Restriction fragments were resolved on a 2% agarose gel.

### *cmv1* fine mapping

Search for recombinants was conducted for successive small intervals on an F2 population from the cross between PS and the NIL SC12-1. DNA was extracted from 100 mg of a young leaf from F_2_ seedlings using the CTAB method^61^. 780 plants were genotyped with the two flanking markers of the *cmv1* interval (CMN61_44 and CMN21_55 (Supplementary Table S1)) and recombinant plants were selected and self-pollinated. 55 F2 recombinant plants were genotyped with a set of internal markers (Supplementary Table S1) in the first interval to determine the exact point of recombination. To position the *cmv1* gene, 20 F_3_ plants from each F_2_ recombinant were inoculated as described above and symptoms were visually scored. A recombinant was considered resistant only when none of the F3 plants showed symptoms. Recombination breakpoints and phenotypic information were combined to narrow the *cmv1* interval. Once the interval was reduced, if several recombinants inside the new interval were available a new set of markers inside the interval (Supplementary Table S1) was used to genotype the recombinants. If no recombinants were available, new F_2_ plants were screened until less than 0.1% of F_2_ plants screened were recombinant.

### Functional analysis of the *cmv1* region

The smallest *cmv1* interval was analyzed using the annotation of the *C. melo* genome version 3.5.1^62^ which is available from the melon genome website (http://melonomics.net/files/Genome/Melon_genome_v3.5.1/). The available gff3 file was used to identify genes and genetic features present in the *cmv1* region. Manual curation of the predicted genes was performed by Blastp of the predicted proteins against the non-redundant protein sequences database (http://blast.ncbi.nlm.nih.gov/Blast.cgi). Polymorphisms between PS and SC lines had been previously detected from re-sequencing data^28, 58^. The Plaza database, version 3.0 dicots (http://bioinformatics.psb.ugent.be/plaza/)^63^, was used to identify orthologues and structure of the candidate genes in other plant species whose genome was available.

### Cloning of *cmv1* candidate gene

The complete coding sequence (CDS) of the *cmv1* candidate from PS and SC was cloned using GATEWAY system (Gateway®Gene Cloning, Thermo Fisher Scientific, MA, USA). Briefly, total RNA was extracted from 100 mg of young leaves using TRI Reagent (Sigma–Aldrich, MO, USA) following manufacturer’s instructions. cDNA was synthesised using SuperScript III Reverse Transcriptase Kit (Invitrogen, CA, USA) and oligo(dT)20 as reverse primer. Complete *cmv1* CDS was obtained by two PCR reactions, using primers pAG06F (ATGGCTCCCATTCTATCGGTA) and pAG23R (CTAATTGATGCAATCTTGAC) for the first 767 nucleotides, of the gene, and pAG22F (GTCAAGATTGCATCAATTAG) with pAG11R (TCAAGTCTTGGAAGCAGCAG) for the remaining 2,136 nucleotides, corresponding to the 3′ part of the coding sequence. These PCR products overlapped 20 base pairs and were assembled in a chimeric PCR to obtain the full-length CDS using primers pAG61F (AC**GTCGAC**TATGGCTCCCATTCTATCGGTA) (incorporating *SalI* restriction enzyme site, in bold) and pAG61R (TC**GATATC**AGTCTTGGAAGCAGCAG) (incorporating *EcoRV* restriction enzyme site, in bold). The chimeric PCR product was inserted between the *SalI* and *EcoRV* sites in pENTR3C (Thermo Fisher Scientific, MA, USA) generating the pENVPS41-PS and the pENVPS41-SC constructs. Constructs were sequenced at Macrogen (Macrogen Europe, Amsterdam, The Netherlands) using primers listed in Supplementary Table S8. Effects of the polymorphisms in the *cmv1* candidate protein were predicted with PROVEAN (http://provean.jcvi.org/index.php)^64^ and MutPred^65^. The binary construct used for melon transformation was made transferring the *CmVPS41PS* allele from pENVPS41-PS to the pEARLY301 binary vector^66^. First, a 1597 bp fragment containing *cmv1* putative UTR and promoter was PCR-amplified from PS genomic DNA using primers AG**GTCGAC**GTTCACTGAGACATTCG and TG**GTCGAC**TGTGATGAACGGCCGATT and cloned into the *SalI* site of pENVPS41-PS, upstream of the starting codon to generate pENProVPS41-PS. Then, pENProVPS41-PS was recombined into the pEARLY301 expression vector^66^, using LR clonase mix enzyme II (Gateway®Gene Cloning, Thermo Fisher Scientific, MA, USA), obtaining the pEX301VPS41-PS, which carries the HA tag sequence at the 3′ end of the gene.

### Determination of the expression levels of *CmVPS41*mRNA by Real Time-PCR

Quantitative Real Time-PCR (qRT-PCR) amplification was performed on three experiments, with three biological replicates each, from SC and PS. 100 mg from three different parts of the leaves (limbo, vein and petiole) were collected from each plant. RNA extraction was carried out using the RNeasy PLANT MINI KIT (Qiagen, Germany) and samples were treated with RNAse free TURBO-DNase I (Applied Biosystem, Ambion, CA, USA) following manufacturer’s instructions. Total RNA was reverse transcribed with SuperScript III Reverse Transcriptase Kit (Invitrogen, CA, USA) and oligo (dT)20 as reverse primer. Quantitative PCR was performed on a LightCycler 480 Real-Time PCR System (Roche Applied Science, IN, USA), using SYBR Green I Mix (Roche Applied Science, IN, USA). Primers TCAACTTCCCGTATTAGTTCCATACA and ACAATGAGTTTGAAGCAAGAGCAAC were designed for *CmVPS41*mRNA amplification using PRIMER EXPRESS® SOFTWARE v 2.0. (Applied Biosystems, CA, USA).Melon cyclophilin (*CmCYP7*)^67^ was used as reference gene. The experiment was done by triplicate. Melting curve analysis at the reaction end-point and no-template controls were used to ensure product-specific amplification and avoid primer-dimer quantification. A five-point standard curve from 10-fold dilutions was built for the cyclophilin reference and *CmVPS41* genes. The efficiency of primers was calculated from the slope of the linear correlation between Cp (Crossing points) and each dilution (E = 10^(−1/slope)^). As the efficiencies between genes did not differ significantly from 1, we used the 2-∆∆Cp method^68^ to calculate the relative expression of *CmVPS41*. Differences were considered significant when fold change was larger than 2.

### Identification and analysis of *CmVPS41* TILLING mutants

A CharMono mutant TILLING collection^34^ was screened for mutants inside the *CmVPS41* coding region. Briefly, pooled DNA from 6,200 M2 families was first PCR amplified with specific primers TAGACTAAAGCTCAAAAGGCTTGCA and GAGTTGTTAGCATACTACATAGGAC, flanking a 732 bp amplicon including exons 5 and 6. This PCR was then used as template in a nested PCR reaction using internal primers carrying a M13 tail (CGACGTTGTAAAACGACGCTATTTCCCATTTCCTC and GATAACAATTTCACACAGGGTCCATGTCATACTCC), plus M13 universal primers, M13F700 (5′-CACGACGTTGTAAAACGAC-3′*)* and M13R800 *(*5′-GGATAACAATTTCACACAGG-3′), labeled at the 5′end with infra-red dyes IRD700 and IRD800 (LI-COR®, Lincoln, NE, USA), respectively. The 574 bp amplicon obtained was used for mutant detection as previously reported^34, 69^. The mutants obtained were subsequently sequenced to identify the nucleotide change. The effect of the observed mutations in the protein was analysedwith PROVEAN (http://provean.jcvi.org/index.php, ref. 64). Families carrying a non-synonymous mutation were selected for phenotyping. Mutant homozygous plants were obtained for each selected family by self-pollinating M2 plants.

### Melon transformation

The plasmid pEX301VPS41-PS was introduced into the AGL0 strain of *Agrobacterium tumefaciens*. Melon transgenic lines were developed by co-cultivation of the Agrobacterium culture with three-days-old cotyledons of SC as previously described^33^. T0 plants that had integrated the transgene were confirmed by PCR. Briefly, after a DNA extraction performed as explained above, the transgene, as well as, the endogenous *CmVPS41* were PCR-amplified with primers ATGGCTCCCATTCTATCG and CACATATTCACCTTCTGTATCA. The endogenous gene produced a 757 bp band, whereas in the transgene, as no introns are present, a 324pb band was amplified. Among the T0 plants that amplified a band of 324 pb, the expression of *CmVPS41PS* allele in leaves was confirmed by RT-PCR. Briefly, total RNA was extracted from 100 mg of young leaves, treated with DNAse and reverse transcribed to cDNA as detailed above, using oligo (dT)20 as reverse primer. Subsequent PCR amplification was carried out with primer ATGTTATTGGAGCACACAGT that anneals with the *CmVPS41*sequence and AGCGTAATCTGGAACATCGT that anneals with the pEX301VPS41-PS, HA tag sequence at the 3′ end of the gene, amplifying a fragment of 642pb (Supplementary Figure S1). The response to CMV-LS and FNY strains was tested in T0 plants expressing the *CmVPS41PS* allele as indicated above. Plants were considered susceptible when symptoms appeared in non-inoculated new leaves.

### Data availability statement

The *C. melo* VPS41 sequence is available at http://melonomics.net.

## Electronic supplementary material

Supplementary figures and tables

Supplementary table S1
