# Supplementary material for: A mutation in the melon Vacuolar Protein Sorting 41prevents systemic infection of Cucumber mosaic virus
Source: Sci Rep. 2017 Sep 5;7:10471. doi: 10.1038/s41598-017-10783-3 (PMC5585375; doi:10.1038/s41598-017-10783-3)
Supplement: Supplementary file 1 — Supplementary figures and tables [file 41598_2017_10783_MOESM1_ESM.pdf]

A mutation in the melon Vacuolar Protein Sorting 41prevents systemic infection of *Cucumber mosaic virus*.

Ana Giner[1+],Laura Pascual[1+!],Michel Bourgeois[1],Gabor Gyetvai[1!!],Pablo Rios[1,!!!],Belén Picó[2], ChristelleTroadec[3],Abdel Bendahmane[3],Jordi Garcia-Mas [1,4], and Ana Montserrat Martín-Hernández[1,4*]

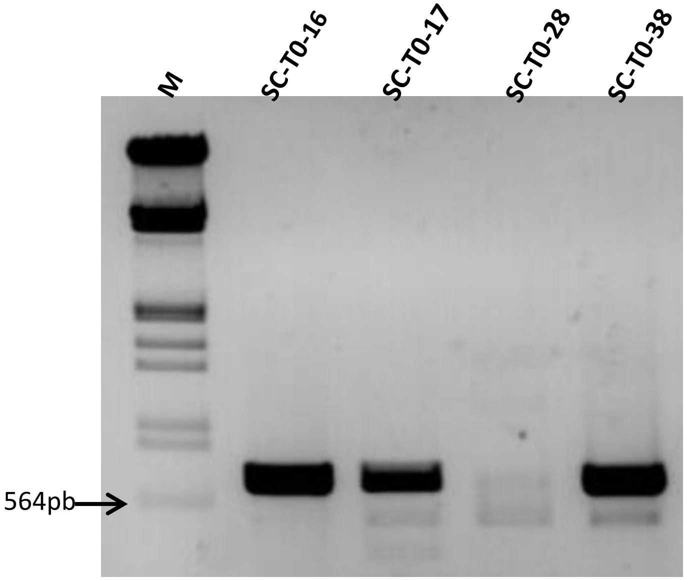

Supplementary Figure S1. Test for expression of *CmVPS41PS* in T0 transgenic plants. RT-PCR results of *CmVPS41PS* in SC-T0 transgenic lines. **M** Lambda DNA/EcoRI+HindIII marker, **SC-T0-16**, **SC-T0-17**, **SC-T0-38**, transgenic lines in which *CmVPS41PS* expression was detected, **SC-T0-28** transgenic line where expression was not detected.

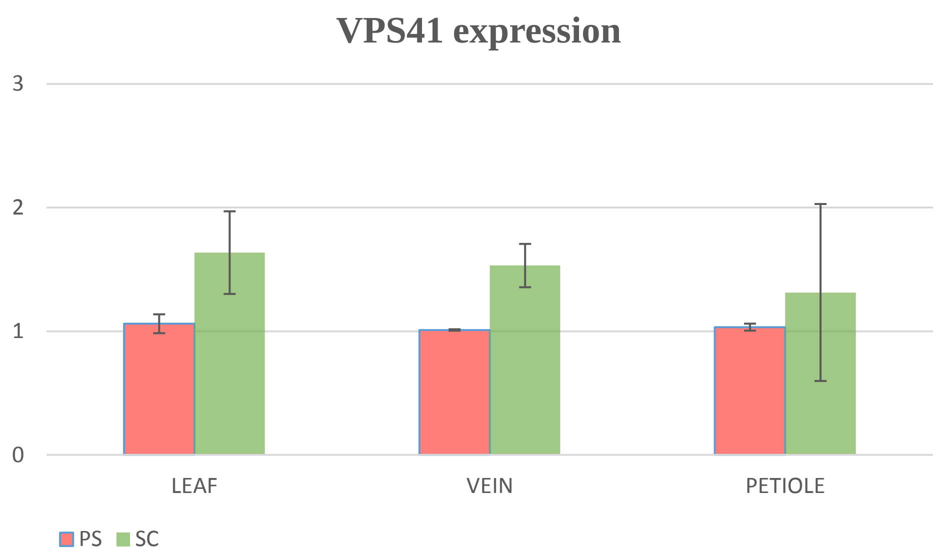

	LEAF		VEIN		PETIOLE	
	**PS**	**SC**	**PS**	**SC**	**PS**	**SC**
Average	**1,0607**	**1,6356**	**1,0101**	**1,5309**	**1,0331**	**1,3133**
Deviation	**0,0763**	**0,3341**	**0,0060**	**0,1753**	**0,0282**	**0,7153**

Supplementary Figure S2. CmVPS41 expression normalized against cyclophilin (CmCYP7) housekeeping gene in leaf, vein and petiole samples. PS susceptible non-inoculated plants, SC resistant plants. Differences of expression between both lines are not statistically significant (fold-change smaller than 2) (in accordance with previous microarray melon data in fruit, where no significant differences were detected (Ref 70).

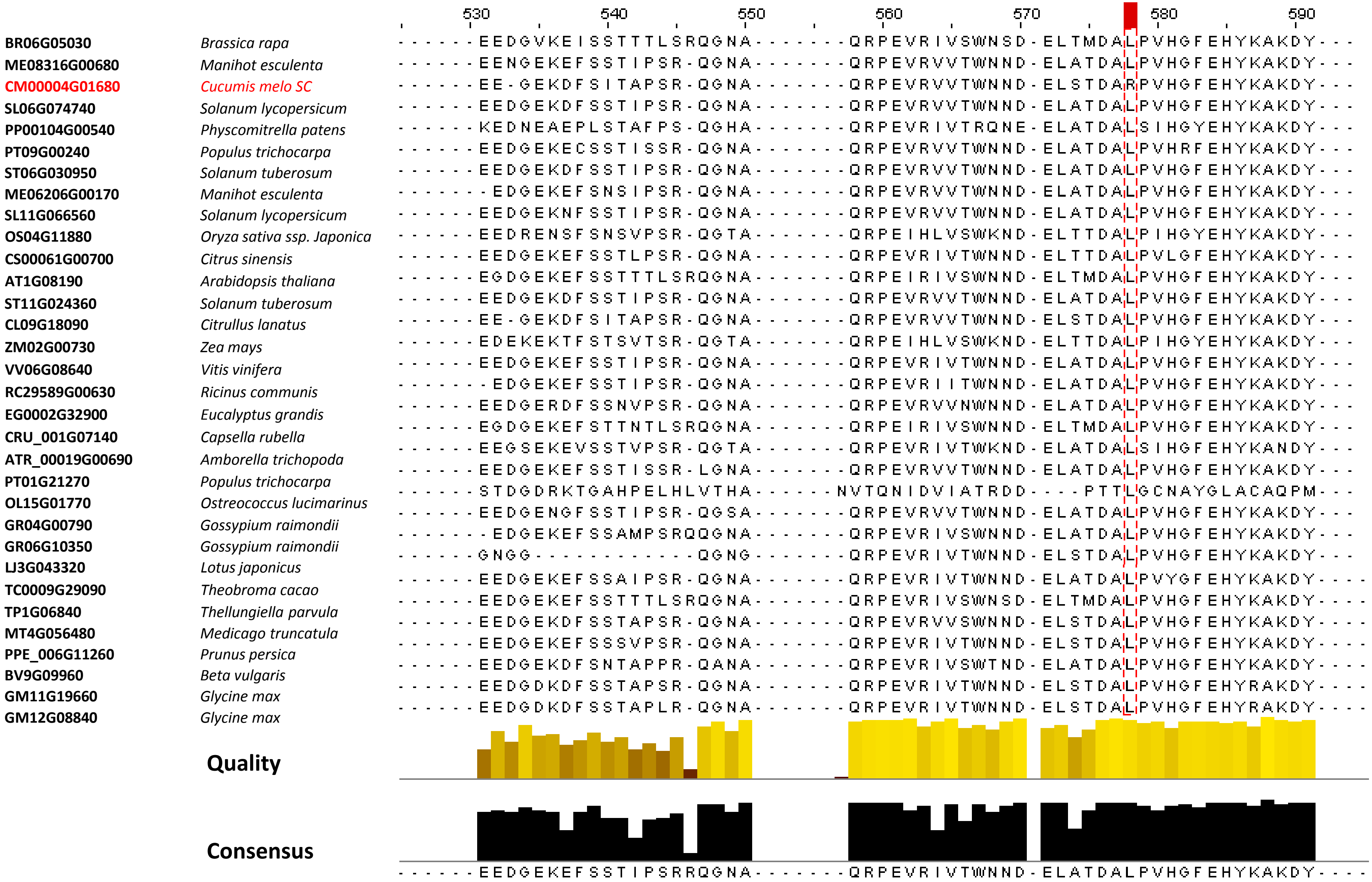

Supplementary Figure S3. Alignment of VPS41 orthologues showing the sequence corresponding to the N-terminal part of *C. melo* exon five, including the causal polymorphism. Only species whose exon five sequence is annotated in PLAZA 3.0 are shown (32 out of 36 orthologues). The red square marks the position of the polymorphism associated with the resistance.

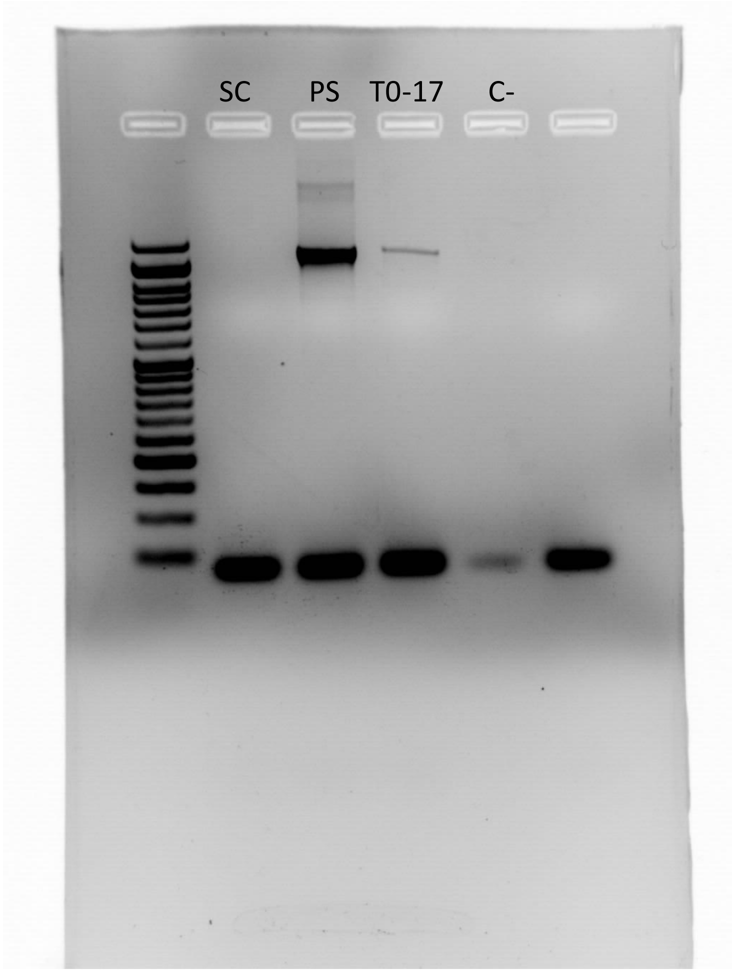

Supplementary figure S4. Full length gel with RT-PCR for detection of CMV-LS shown in Figure 3a

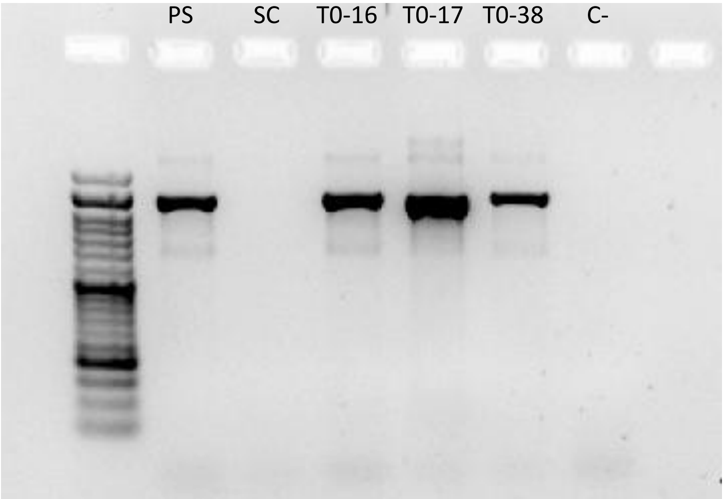

Supplementary figure S5. Full length gel with RT-PCR for detection of CMV-FNY shown in figure 3b.

Table S2. Predicted genes within the smallest *cmv1* interval

Chr	Start	End	Sense	ID	Size (bp)	Number of ESTs	Description
chr12	9205072	9207048	-	ID=MELO3C004835	1976	4	Similar to Lysosomal Pro-X carboxypeptidase (Homo sapiens) (uniprot_sprot:sp|P42785|PCP_HUMAN)
chr12	9223896	9224372	+	ID=MELO3C004834	476	0	Unknow function
chr12	9233256	9245726	-	ID=MELO3C004833	12470	1620	Similar to Crooked neck-like protein 1 (Homo sapiens) (uniprot_sprot:sp|Q9BZJ0|CRNL1_HUMAN)
chr12	9261314	9261721	+	ID=MELO3C004832	407	0	Unknow function
chr12	9308238	9309629	+	ID=MELO3C004831	1391	209	Similar to Vacuolar protein sorting-associated protein 41 homolog (Solanum lycopersicum) (uniprot_sprot:sp|P93231|VPS41_SOLLC)
chr12	9310628	9311002	-	ID=MELO3C004830	374	0	Similar to Putative uncharacterized protein (Vitis vinifera) (uniref90:UniRef90_A5BWU8)
chr12	9311921	9312238	-	ID=MELO3C004829	317	0	Similar to Gag-pol polyprotein, putative (Asparagus officinalis) (uniref90:UniRef90_Q2A9Z5)
chr12	9313543	9313824	-	ID=MELO3C004828	281	0	Unknow function
chr12	9316976	9337164	+	ID=MELO3C004827	20188	2266	Similar to Vacuolar protein sorting-associated protein 41 homolog (Solanum lycopersicum) (uniprot_sprot:sp|P93231|VPS41_SOLLC)

Table S3. *CmVPS41* ORF. Predicted exons for both *CmVPS41* predicted genes in the annotation of *C. melo* version 3.5.1 and manual annotations on the same version 3.5.1.

**Predicted complete gene**			**Manually annotated ORF**		
**Exon**	**Start**	**End**	**Exon**	**Start**	**End**
MELO3C004831T1.exon1	9308238	9308625	CmVPS41.exon1	9308365	9308625
MELO3C004831T1.exon2	9309056	9309325	CmVPS41.exon2	9309056	9309325
MELO3C004831T1.exon3	9309423	9309629	CmVPS41.exon3	9309423	9309602
MELO3C004827T1.exon1	9316976	9316990	Not present		
MELO3C004827T1.exon2	9317278	9317541	CmVPS41.exon4	9317278	9317541
MELO3C004827T1.exon3	9318229	9318361	CmVPS41.exon5	9318229	9318361
MELO3C004827T1.exon4	9318450	9318540	CmVPS41.exon6	9318450	9318540
MELO3C004827T1.exon5	9319045	9319147	CmVPS41.exon7	9319045	9319147
MELO3C004827T1.exon6	9320215	9320318	CmVPS41.exon8	9320215	9320318
MELO3C004827T1.exon7	9320389	9320482	CmVPS41.exon9	9320389	9320482
MELO3C004827T1.exon8	9320579	9320734	CmVPS41.exon10	9320579	9320734
MELO3C004827T1.exon9	9320814	9320876	CmVPS41.exon11	9320814	9320876
MELO3C004827T1.exon10	9320978	9321043	CmVPS41.exon12	9320978	9321043
MELO3C004827T1.exon11	9327401	9327601	CmVPS41.exon13	9327401	9327601
MELO3C004827T1.exon12	9334583	9334660	CmVPS41.exon14	9334583	9334660
MELO3C004827T1.exon13	9334732	9334851	CmVPS41.exon15	9334732	9334851
MELO3C004827T1.exon14	9334983	9335063	CmVPS41.exon16	9334983	9335063
MELO3C004827T1.exon15	9335143	9335228	CmVPS41.exon17	9335143	9335228
MELO3C004827T1.exon16	9336178	9336259	CmVPS41.exon18	9336178	9336259
MELO3C004827T1.exon17	9336345	9337164	CmVPS41.exon19	9336345	9336794

Table S4. TILLING mutants detected in a genomic fragment including CmVPS41 exons 5 and 6.

Family	Original codon	Mutated codon	AA change	Position	Possition from the ORF start	effect PROVEAN	effect PROVEAN
411	GAG	GAA	E354E	EXON	-	-	-
451	-	-	-	INTRON	-	-	-
5857	UCU	UAU	S344Y	EXON	1031	-4 252	Deleterious
6190	-	-	-	INTRON	-	-	-
1326	GGU	AGU	G375S	EXON	1110	-0.857	Neutral
1410	-	-	-	INTRON	-	-	-
1536	GAA	AAA	E332K	EXON	994	-3 253	Deleterious
1736	-	-	-	INTRON	-	-	-
3425	GAC	AAC	D360N	EXON	1078	-4 736	Deleterious
3487	-	-	-	INTRON	-	-	-
2291	-	-	-	INTRON	-	-	-
3932	-	-	-	INTRON	-	-	-
4895	AGA	AAA	R330K	EXON	989	-2 393	Neutral
6331	-	-	-	INTRON	-	-	-

Table S5. Predicted effects of the *CmVPS41* changes between PS/SC using PROVEAN and MutPred software

**Genome possition**		**9317350**	**9318296**	**9327473**
**Possition from ORF start**		**784**	**1043**	**1858**
**PS allele**		**C**	**T**	**T**
**SC allele**		**G**	**G**	**C**
**aa Change**		**P262A**	**L348R**	**S620P**
**Provean predictions**				
**Score**		**-0,903**	**-5,929**	**1,203**
**Predicted effects**		**Neutral**	**Deleterious**	**Neutral**
**MutPred predictions**				
**Probability of deleterious mutation**		**0,33**	**0,801**	**0,572**
**MOLECULAR MECHANISM DISRUPTED**	Actionable Hypotheses	Na	Na	Gain of catalytic residue at P619 (P = 0.0123)
	Confident Hypotheses	Na	Loss of stability (P = 0.0121) Loss of loop (P = 0.0203) Gain of disorder (P = 0.0401)	Na
	Very Confident Hypotheses	Na	Loss of sheet (P = 0.007)	Na

Table S6. C. melo accessions and genotyping results. Accession name, code (according to previous studies (Refs. 35 and 36) and origin is given for each accession. Accessions are classified according the C. melo subspecies (ssp. melo and ssp. agrestis) and cultivar group (inodorus, cantalupensis, reticulatus, ameri group (including ameri, adana and chandalack), flexuosus (including chate) and dudaim, within ssp. melo, and momordica, conomon (including chinensis, conomon and makuwa), tibish, chito and agrestis, within ssp. agrestis.

Local/commercial name	Code	Origin	CmVPS41		
			SNP/CAP/TseI	SNP/CAP/BsiEI	SNP/seq
			G/C Exon4	G/T Exon5	C/T Exon13
C.melo* ssp *melo					
Inodorus commercial cultivars					
Piel de sapo Piñonet	In-PsPiñSp	Spain	SC	PS	SC
Honeydew green flesh	In-HoneyDewUSA	USA	SC	PS	SC
Inodorus Spanish landraces					
Pipa de Oro	In-PsPipaSp	Spain	SC	PS	SC
Piñonocillo	In-PsPiñonSp	Spain	SC	PS	SC
Caña Dulce	In-AmCañSp	Spain	SC	PS	SC
Amarillo Oro	In-AmAoroSp	Spain	SC	PS	SC
Blanco Escrito	In-BBescrSp	Spain	SC	PS	SC
Mollerusa	In-TeMollSp	Spain	SC	PS	SC
Largo de Villaconejos	In-TeLVillSp	Spain	SC	PS	SC
Coca	In-LaCocaSp	Spain	SC	PS	SC
Melon de Calamonte	In-LaCalSp	Spain	SC	PS	SC
Madura Amarilla	In-MAmSp	Spain	SC	PS	SC
Inodorus landraces from Europe, North Africa and Asia					
Kirkagac	In-KirkTur	Turkey	SC	PS	SC
Maazoon	In-MaazTun	Tunisia	SC	PS	SC
Baskavas	In-BaskGreece	Greece	SC	PS	SC
Hami melon	In-HamiChi	China	SC	PS	SC
Asli	In-AsliTun	Tunisia	SC	PS	SC
Ameri/adana/chandalack and other landraces from Europe and Asia					
Korça	Am-KorcaRus	Russia	SC	PS	PS
Kizil Uruk	Am-Kizil-Uzbe	Uzbequistan	SC	PS	SC
Mucha Nesvi*	Am-NesviGeor	Georgia	SC	PS	SC
Chandalack (PI 276660)	Am-ChandAfg	Afghanistan	SC	PS	SC
Ananas Yokneam*	Am-YokIs	Israel	SC	PS	SC
Zatta	La-Zat-Ita	Italy	SC	PS	SC
Cantalupensis and *reticulatus* commercial cultivars and landraces from Europe, Asia, and America					
Cantalup D´Alger*	Can-CAFran	France	SC	PS	SC
Dulce	Can-DulUSA	USA	SC	PS	SC
Nantais Oblong*	Can-NOFran	France	PS	PS	PS
Noir des carmes	Can-NCFran	France	PS	PS	PS
PMR-45	Can-PMRUSA	France	SC	PS	PS
Prescott fond blanc	Can-PresFran	France	PS	PS	PS
Vedrantais	Can-VedFran	France	PS	PS	PS
WI_998	Can-WiUSA	USA	SC	PS	SC
Y285	Can-Y	Yugoslavia	SC	PS	Het
Ar Hale's Best Jumbo*	Can-HBJUSA	USA	SC	PS	SC

Earl´s Favourite*	Can-EfJa	Japan	SC	PS	SC
Old Time Tenesse*	Can-TeUSA	USA	SC	PS	SC
Persian Small Type*	Can-PSUSA	USA	SC	PS	SC
Seminole	Can-SemUSA	USA	SC	PS	SC
Flexuosus and *chate* accessions from Asia and Europe					
Snake melon	Flex-SnakeSA	Saudi Arab.	SC	PS	SC
Arya	Flex-AryaInd	India	SC	PS	SC
Carosello	Chate-CarIta	Italy	SC	PS	SC
Dudaim accession					
Qeen's pocket melon*	Dud-QPMAfg	Afganistan	SC	PS	SC
C.melo* ssp.*agrestis					
Momordica accessions from India					
PI 124112	Mom-PI124Ind	India	SC	PS	SC
MR1	Mom-MR1Ind	India	SC	PS	SC
PI 414723	Mom-PI414Ind	India	SC	PS	SC
Conomon accession from Far East					
Ginsen makuwa	Con-GMJa	Japan	SC	SC	SC
Freeman's Cucumber	Con-FreeCJa	Japan	SC	PS	SC
Paul*	Con-PauPol	Poland	SC	PS	SC
Tibish, chito and *agrestis* from Asia and Africa					
Tibish Khurtagat*	Tibish-KSud	Sudan	SC	PS	SC
Velleri (PI 164320)	Chi-VellInd	India	SC	PS	SC
PI 185111	Ag-15591Gha	Ghana	SC	PS	SC
Tayer	Ag-TayCam	Cameroon	SC	PS	SC
Trigonus	Ag-TriInd	India	SC	PS	SC

This collection of accessions is currently maintained at the Institute for the Conservation and Breeding of the Agrobiodiversity (COMAV) genebank (www.comav.upv.es). Accessions marked with an asterisk were kidly supplied by M.Pitrat (INRA). PI accessions are from USDA-NPGS gene bank.

Table S7. *VPS41* orthologue genes in sequenced plant species according to PLAZA3.0

Gene_id	species	short name species	chr
AT1G08190	Arabidopsis thaliana	ath	1
ATR_00019G00690	Amborella trichopoda	atr	scaffold00019
BR06G05030	Brassica rapa	bra	A06
BV9G09960	Beta vulgaris	bvu	Bvchr9.sca018
CL09G18090	Citrullus lanatus	cla	Chr9
CM00004G01680	Cucumis melo	cme	CM3.5_scaffold00004
CP00047G00240	Carica papaya	cpa	supercontig_47
CR03G15610	Chlamydomonas reinhardtii	cre	chromosome_3
CRU_001G07140	Capsella rubella	cru	scaffold_1
CS00061G00700	Citrus sinensis	csi	scaffold00061
EG0002G32900	Eucalyptus grandis	egr	scaffold_2
GM11G19660	Glycine max	gma	Gm11
GM12G08840	Glycine max	gma	Gm12
GR04G00790	Gossypium raimondii	gra	Chr04
GR06G10350	Gossypium raimondii	gra	Chr06
LJ3G043320	Lotus japonicus	lja	chr3
MD00G253560	Malus domestica	mdo	MDC010900.373
MD00G452170	Malus domestica	mdo	MDC020832.278
ME06206G00170	Manihot esculenta	mes	scaffold06206
ME08316G00680	Manihot esculenta	mes	scaffold08316
MT4G056480	Medicago truncatula	mtr	chr4
OL15G01770	Ostreococcus lucimarinus	olu	Chr_15
OS04G11880	Oryza sativa ssp. japonica	osa	4
PP00104G00540	Physcomitrella patens	ppa	scaffold_104
PPE_006G11260	Prunus persica	ppe	scaffold_6
PT01G21270	Populus trichocarpa	ptr	Chr01
PT09G00240	Populus trichocarpa	ptr	Chr09
RC29589G00630	Ricinus communis	rco	29589
SL06G074740	Solanum lycopersicum	sly	ch06
SL11G066560	Solanum lycopersicum	sly	ch11
ST06G030950	Solanum tuberosum	stu	chr06
ST11G024360	Solanum tuberosum	stu	chr11
TC0009G29090	Theobroma cacao	tca	scaffold_9
TP1G06840	Thellungiella parvula	tpa	ch1-1
VV06G08640	Vitis vinifera	vvi	chr6
ZM02G00730	Zea mays	zma	2

Table S8. Primers used for sequencing , covering the complete sequence of *cmv1 cDNA* .

Primer	Sequence 5'	Lenght	PCR TM.ºC
pAG 049	CACATATTCACCTTCTGTATCA	22	56
pAG 042	TGGCTAGGATACAAAGACCA	20	56
pAG 022	GTCAAGATTGCATCAATTAG	20	52
pAG 023	CTAATTGATGCAATCTTGAC	20	52
pAG 024	TCATACTGCCCATCTACGA	19	55
pAG 041	GTGTATAATGTTGACTACTACG	22	56
pAG 025	ATGTTATTGGAGCACACAGT	20	54
pAG 011	TCAAGTCTTGGAAGCAGCAG	20	56
